# Longitudinal Changes in Functioning and Disability in Patients with Disorders of Consciousness: The Importance of Environmental Factors

**DOI:** 10.3390/ijerph120403707

**Published:** 2015-04-01

**Authors:** Michelle Willems, Davide Sattin, Ad J.J.M. Vingerhoets, Matilde Leonardi

**Affiliations:** 1 Neurology, Public Health, Disability Unit—Scientific Directorate, Neurological Institute Carlo Besta Istituto di Ricovero e Cura a Carattere Scientifico (IRCCS) Foundation, Via Celoria 11, Milan 20133, Italy; E-Mails: davide.sattin@istituto-besta.it (D.S.); leonardi@istituto-besta.it (M.L.); 2 Department of Medical & Clinical Psychology, Tilburg University, P.O. Box 90153, Tilburg 5000 LE, The Netherlands; E-Mail: vingerhoets@uvt.nl

**Keywords:** unresponsive wakefulness syndrome, vegetative state, minimally conscious state, disorders of consciousness, disability, functioning, International Classification of Functioning, Disability, and Health, longitudinal study

## Abstract

Disorders of consciousness are neurological conditions associated with low levels of functioning which pose a serious challenge to public health systems. The current study aimed to examine longitudinal changes in functioning in patients with disorders of consciousness and to identify associated biopsychosocial factors using the International Classification of Functioning, Disability, and Health. An Italian sample of 248 patients was assessed longitudinally. Differences in relative variability (an index of change that controls for baseline levels) between acute and chronic patients and predictors of relative variability in “*Activities* & *Participation*” were examined. Results showed that there were subgroups of patients whose functioning improved over time. The number of problems in “*Activities* & *Participation*” decreased in acute patients over time, whereas in chronic patients, an increase was found. The significant difference in relative variability for the environmental factor “*support and relationships*” reflects the increase in facilitators in acute patients, whereas the number of facilitators in chronic patients remained unchanged over time. Age at event, time from event, and relative variability in “*Environmental Factors*” were significant predictors of relative variability in “*Activities* & *Participation*”. It is of clinical relevance that patients with disorders of consciousness are kept in a supportive and facilitative environment, in order to prevent a decline in their functioning. Moreover, caregivers should receive tailored support in order to enhance and facilitate appropriate care of patients with disorders of consciousness.

## 1. Introduction

Disorders of consciousness include unresponsive wakefulness syndrome and minimally conscious state. Unresponsive wakefulness syndrome [1], or “*wakefulness without awareness*” [2], is a post-coma syndrome in which the patient shows no signs of awareness of the self or the environment; no sustained, reproducible, purposeful, or voluntary behavioral responses to visual, auditory, tactile, or noxious stimuli; and no indication of language comprehension or expression. However, the hypothalamic and brainstem autonomous functions, and the sleep-wake cycle are retained [3]. Unresponsive wakefulness syndrome has often been referred to as “*vegetative stat*e”. However, due to the negative connotations associated with this term [1], we have chosen to use the recently introduced term “*unresponsive wakefulness syndrome*” in the remainder of this article. Minimally conscious state, on the other hand, is a post-coma condition in which the patient has recovered to a state of poor and inconsistent responsiveness to stimuli, but shows limited evidence of awareness of themselves and their surroundings [4,5,6]. 

Unresponsive wakefulness syndrome and minimally conscious state can be either acute and reversible phases or chronic and irreversible conditions [2]. Patients with disorders of consciousness demonstrate low levels of functioning and require high levels of medical and nursing care for extended periods of time [7]. In fact, the clinical course of chronic disorders of consciousness often occupies many years or even decades [8], posing a significant demand on health care systems [9]. Moreover, studies have shown that, in recent years, the number of patients with disorders of consciousness is increasing [10,11,12], presumably caused by an increasing incidence of stroke [13], a decreasing mortality rate after stroke [14,15], and advances in emergency medical treatment and critical care leading to an increased survival of patients with disorders of consciousness [12,16]. As such, unresponsive wakefulness syndrome and minimally conscious state pose a serious challenge to the public health systems [17,18], which has urgently called for longitudinal studies to be conducted to analyze functioning and disability in these patients over time. 

Over the past few decades, the public health approach to disability has shifted away from the medical model via the social model towards the biopsychosocial model of functioning and disability [19]. According to this model, disability can be defined as “*a difficulty in functioning at the body, person, or societal levels, in one or more life domains, as experienced by an individual with a health condition in interaction with contextual factors*” [20]. This approach to disability is reflected by the World Health Organization’s International Classification of Functioning, Disability, and Health (ICF) [21], which provides a standard language and a common framework for the description of health and health-related domains and their interaction with the environment. 

The ICF enables the collection of data in four different, but related, domains: Body Functions, Body Structures, Activities & Participation, and Environmental Factors. Body Functions are the physiological functions of body systems (e.g., b140 Attention functions). Body Structures are anatomical parts of the body, such as organs, limbs and their components (e.g., s110 Structure of the brain). Activities & Participation comprise the full range of activities that individuals undertake, from the simple and personal (e.g., d510 Washing oneself) to the complex and socially constructed (e.g., d720 Complex interpersonal interactions). Finally, Environmental Factors represent the physical (e.g., e225 Climate), social (e.g., e320 Friends), and attitudinal environment (e.g., e465 Social norms, practices, and ideologies) in which persons live and conduct their lives. By comprising information on both the health and the environmental aspects of functioning and on their interaction causing disability, the ICF provides a complete and comprehensive picture of an individual’s level of functioning and disability, enabling clinicians to target interventions that improve functioning as well as interventions directed either at the person or at the individual’s environment [19]. Therefore, ICF has become the instrument of choice for the collection of public health data on functioning and disability.

Recently, several Italian studies have investigated functioning and disability in patients with disorders of consciousness using the ICF. An ICF-based description in 21 patients with unresponsive wakefulness syndrome revealed that the prevalence of impairments in the Body Structures domain was relatively limited, whereas within the Environmental Factors domain, a high number of facilitators were found that had a positive effect on problems mainly related to mobility and self-care [22]. A study by Leonardi and colleagues [23] which aimed to identify relevant ICF categories in 36 children and adolescents with disorders of consciousness concluded that the ICF is a useful instrument to describe functioning and disability in this population. Moreover, a study by Sattin and colleagues [7], that compared functioning and disability between 396 patients with unresponsive wakefulness syndrome and 168 patients in minimally conscious state concluded that their functioning and disability profiles were rather similar, indicating that both groups of patients require similarly high levels of care and assistance. Recently, a specific ICF checklist for patients with disorders of consciousness (International Classification of Functioning, Disability, and Health-Disorders Of Consciousness; ICF-DOC) was developed [24], containing 37 categories from Body Functions domain, 13 categories from the Body Structures domain, 46 from Activities & Participation domain, and 31 categories from the Environmental Factors domain (a complete overview of the ICF-DOC is presented in Table S1 of the Supplementary Information) . It was concluded that the new ICF-DOC checklist is a useful instrument to collect functioning and disability data in adult patients with disorders of consciousness and to monitor changes in functioning and disability over time.

To date, however, few longitudinal studies on adult patients with disorders of consciousness using the ICF are available. Although there have been several longitudinal studies that examined functioning in patients with disorders of consciousness [25,26,27,28,29,30], of which most [25,26,27,28,30] had a longer follow-up than that of the current study, and some [27,28,30] conducted multiple follow-up assessments, these studies made use of global instruments to measure disability and functioning, such as (predominantly) the Disability Rating Scale, Post-Acute Level Of Consciousness scale, Glasgow Outcome Scale (Extended), Coma Recovery Scale-Revised, Glasgow Coma Scale, and the Functional Independence Measure. Although these instruments provide relevant information in relation to the level of consciousness, basic cognitive and motor skills, and, at best, activities of daily living, they do not offer a more detailed description of patients in terms of their capabilities and impairments [28], nor do they take into account factors from the patient’s environment. In addition, sample sizes of all these studies were small (<100), with the exception of one study [30], and some of these studies had a retrospective study design [25,27,28]. Furthermore, some studies were conducted on children and young adults [25,26], or comprised only of patients whose disorder of consciousness resulted from traumatic aetiology [26,30], both of which groups are known to have a better prognosis in comparison to adult patients and those with non-traumatic aetiology, respectively. A recent study by Seel *et al.* [31] is especially interesting, as it describes and evaluates a comprehensive early treatment program for patients with disorders of consciousness and their families based upon the components of the ICF. However, this was a single-centre, retrospective study in which patients had predominantly traumatic aetiology. Moreover, this study only evaluated changes on a selection of ICF chapters and did not examine factors related to these changes. 

Therefore, the aims of the current prospective study are (1) to examine longitudinal changes in functioning and disability using the ICF in a large, representative sample of patients with unresponsive wakefulness syndrome or in minimally conscious state and (2) to detect biopsychosocial correlates that are associated with these changes. 

## 2. Materials and Methods

### 2.1. Study Design and Population

The current study is an observational, longitudinal, multi-center study conducted in ninety Italian centers specializing in post-acute treatment or long-term assistance and care of patients with disorders of consciousness. The project was coordinated by the Neurological Institute “*Carlo Besta*” Foundation. 

The first wave of data collection (T0) was done within the “*National Study on Functioning and Disability in Vegetative and in Minimal Conscious State Patients*”, and was conducted between June 2009 and March 2010. Enrolled in the study were adult patients with disorders of consciousness who resided in the various participating centers, as well as patients who resided at home and were regularly followed up by specialists from the participating centers. All patients had been diagnosed with unresponsive wakefulness syndrome or minimally conscious state by qualified medical doctors with expertise in disorders of consciousness, according to the internationally recognized diagnostic criteria proposed by the Aspen Neurobehavioural Conference Workgroup [4]. Patients were excluded from participation in the study if they had been diagnosed with other neurological and psychiatric disorders prior to the acute event, if their unresponsive wakefulness syndrome or minimally conscious state diagnosis was uncertain, or if their legal representatives did not provide consent to participate. The second wave of data collection (T1) was carried out within the Italian national project “*PRECIOUS*” and was conducted between July 2011 and September 2012. This study was part of a broader project that aimed to collect epidemiological and clinical data on patients with disorders of consciousness in order to highlight the complex condition of these cases, and to develop better management strategies and inclusive health care programs.

Both studies were conducted in accordance with the Declaration of Helsinki and had been approved by Neurological Institute “*Carlo Besta*” Foundation’s Ethics Committee (Project Identification Codes DGPREV/P/145021/F.3.a.d/282, approval date 9 December 2009 (T0), and DGPREV/I/F.3.a.d/2010/411, approval date 12 November 2010 (T1) and were ratified by the other participating centers. Written informed consent to participate in the study was obtained from the patients’ legal representatives and data were anonymized to protect patients’ privacy. 

### 2.2. Procedure

The study protocol comprised of demographical and clinical questionnaires and an adapted ICF checklist for patients with disorders of consciousness. Each participating center collected information on adult patients with disorders of consciousness who were residing either within their units or at home (patients with periodic medical controls in the hospital). To ensure the quality of the data collected by the different centers, the coordinating center provided a formal training to all professionals involved in data collection. Cases where parts of the data were missing were controlled for in the data and, when possible, completed. For a number of ICF categories, the scores were cross-validated with other measures (e.g., values on b110 Conscious functions were checked using the patient’s diagnosis). Where necessary, adjustments were made. Overall, the quality of the data was good. Nine cases were excluded due to the presence of too many missing values (*i.e*., >25% on any of the four ICF domains). 

### 2.3. Materials

*Demographical and clinical questionnaires:* Information on gender, age at acute event, place of residence and time from event was collected through *ad hoc* questionnaires that were developed during the “*National Study on Functioning and Disability in Vegetative and in Minimal Conscious State Patients*” project.

*Functioning, Disability, and Health* Data were collected using the ICF-DOC checklist [24]. The Body Functions domain consisted of 35 individual ICF categories clustered within eight overarching chapters. The Body Structures domain comprised 13 individual ICF categories pertaining to seven chapters. The Activities & Participation domain contained 45 ICF categories grouped within nine chapters. For this domain, the individual ICF categories were rated twice, assessing the patient’s *Performance* and *Capacity* in terms of activities and participation. In line with the ICF manual, the Performance qualifier describes what activities an individual undertakes in his or her current environment, whereas the Capacity qualifier represents an individual’s intrinsic ability to perform a task or action. This construct indicates the highest possible level of functioning that the individual can achieve in a given domain at a given time. The ICF domain on Environmental Factors consisted of 31 individual ICF categories clustered into five chapters. As environmental factors may either serve as a facilitator or as a barrier to a person’s functioning, depending on the individual patient, ratings on each ICF category were, according to the ICF guidelines, transformed into two variables. Rating as a *Facilitator* indicates that a certain environmental factor has a positive influence on the functioning of the person, whereas rating as a *Barrier*, on the other hand, implies that the environmental factor has a hindering effect on the person’s functioning. 

The generic severity qualifier, which applies to all ICF categories, was rated on a five-point scale ranging from 0–4, with 0 = no problem, 1 = mild problem, 2 = moderate problem, 3 = severe problem, and 4 = complete problem. Likewise, the rating of facilitators and barriers was 0 = no facilitator/barrier, 1 = mild facilitator/barrier, 2 = moderate facilitator/barrier, 3 = substantial facilitator/severe barrier, and 4 = complete facilitator/barrier. If a problem was detected, but adequate information as to its severity was lacking, qualifier 8 (not specified) was applied.

### 2.4. Statistical Analyses

Descriptive statistics were used to illustrate socio-demographic characteristics of the study participants. Continuous variables were presented as means and standard deviations and categorical variables as frequencies and percentages.

Scores on the individual ICF categories were transformed into indexes of extension and severity [7] for the various ICF chapters within the four ICF domains. The extension index comprised of the number of categories within an ICF chapter with a score of 1–4 or 8, indicating the presence of a problem of any severity. Likewise, the severity index comprised of the number of categories with a score of 3–4, indicating the presence of severe and complete problems. Lastly, an index was created comprising of the number of categories with a score of 8, indicating the presence of a problem of unspecified severity. Due to its ambiguous nature, this index was only used for descriptive purposes and was not included in the statistical analyses. To account for the different number of categories within each ICF chapter, extension and severity indexes were divided by the number of categories within the chapters and multiplied by 100, resulting in transformed scores ranging from 0–100 for every extension and severity index. 

For every ICF chapter, difference scores were computed by subtracting the transformed extension and severity indexes at T0 from those at T1 (T1–T0). Subsequently, these difference scores were trichotomized: scores < 0 were considered an improvement (*i.e*., a decrease of problems), a score of 0 indicated stability (*i.e*., the number of problems remained the same), and scores >0 represented deterioration in functioning (*i.e*., an increase in the number of problems). Frequencies and percentages of the number of patients who improved, remained stable, and deteriorated with respect to their scores on the various chapters within the four ICF chapters were computed. The number of patients included in the analysis, which varied due to missing values on the individual ICF categories, was reported in all tables. 

Subsequently, we compared the level of change in the Body Functions and Activities & Participation domains and chapters of the Environmental Factors domain in acute *vs.* chronic patients. Patients were considered “*acute*” if they were for less than one year fulfilling criteria for unresponsive wakefulness syndrome or minimally conscious state at the time of the T0 assessment, whereas patients were considered “*chronic*” if they were displaying characteristics of unresponsive wakefulness syndrome or minimally conscious state for more than one year [32]. A disadvantage in examining change using absolute difference scores (T1–T0) is that they do not take into account the baseline level. For example, a patient who has a score 4 at T0 and T1, and a patient who has a score 0 at T0 and T1, both have an absolute difference score of 0, while the first patient had the possibility to improve 4 points on the scale and the second patient could only deteriorate or retain the same score. Therefore, in order to account for differences at T0 that influence the potential level of change, an index of relative variability was calculated using the formula [(*b* – *a*)/*a*)] × 100, *i.e.*, by subtracting the transformed extension index at T0 from that at T1, dividing the result by the score at T0, and multiplying by 100 [33]. Given the non-normality of the relative variability index, differences between acute and chronic patients were examined using the non-parametric Mann Whitney *U* test. Bonferroni correction was applied to control for Type I errors.

Finally, the relationships between relative variability in the total score on the Activities & Participation domain (extension, performance) and age at acute event, time from event (assessed at T0), and relative variability in the Body Functions and Environmental Factors domain were examined by linear regression analysis. Evaluation of assumptions showed that relative variability in Activities & Participation, relative variability in Body Functions, and time from event were not normally distributed. Therefore, a logarithmical transformation log 10 [(max + 1) – *x*] was applied to the first two variables and an inverse transformation 1/[(max + 1) – *x*] was applied to the time for an event variable in order to obtain a normal distribution.

In all analyses, hypothesis testing was two-tailed and *p*-values < 0.05 were considered to indicate statistical significance. Data were analyzed using SPSS version 18.0 (SPSS Inc., Chicago, IL USA). With respect to the Mann Whitney analyses, the effect size Cohen’s *d* was calculated using G*Power software and interpreted as ≤ 0.19 = marginal, 0.20–0.49 = small, 0.50–0.79 = medium, and ≥ 80 = large. 

## 3. Results

**Figure 1:**
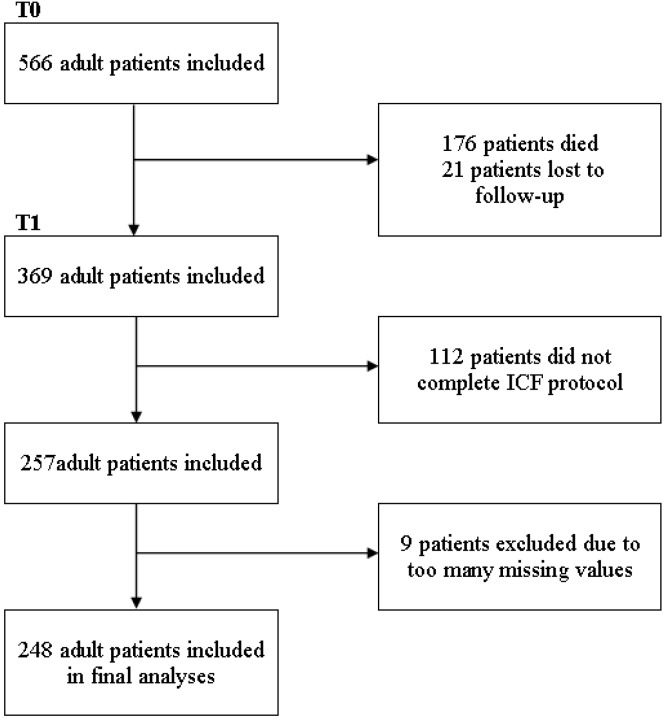
This flow chart presents the number of patients initially included at T0 and the final number of patients included in the longitudinal analyses, providing reasons for exclusion and drop-out.

A flow chart of patient inclusion is shown in Figure 1. In total, 248 patients had complete socio-demographic and ICF data available on both measurement occasions, and these cases were included in the final analyses. The mean follow-up duration between T0 and T1 was 30.21 months (SD 2.85, range 22–40). 

The socio-demographic characteristics of the sample are presented in Table 1. The sample contained a slight preponderance of male patients (54.8%). The mean age at the acute event was 49.19 years. The mean time from event at T0 was 3.98 years. At both T0 and T1, the majority of patients resided in long-term care facilities. Only a small proportion (5.2%) of patients resided at home. Between T0 and T1, almost all patients were involved in one or multiple types of rehabilitation, such as mobilization, muscular stretching, passive musculoskeletal exercises, physical therapy, nursing, respiratory exercises, speech rehabilitation, psychological rehabilitation, and/or music therapy. 

**Table 1:** Socio-demographic characteristics of the study sample.

Socio-Demographic Characteristics	*N*	%	M	SD	Min	Max
Age at event (years)			49.19	16.98	8.39	85.14
Time from event at T0 (years)			3.98	3.41	0.08	23.42
Gender						
Male	136	54.8				
Female	112	45.2				
Residence at T0						
Post-acute	41	16.5				
Long-term care	199	80.2				
Domicile	8	3.2				
Residence at T1						
Post-acute	33	13.3				
Long-term care	202	81.5				
Domicile	13	5.2				

Note: Continuous variables are represented as means, standard deviations, and minima and maxima. Categorical variables are represented as frequencies and percentages.

### 3.1. Improvement, Stability, and Deterioration of the International Classification of Functioning, Disability, and Health Chapters between T0 and T1

The numbers and percentages of patients whose functioning improved, remained stable, or deteriorated with respect to the extension and severity indexes of the four ICF domains are presented in Table 2, Table 3, Table 4 and Table 5. Strikingly, in the whole sample, there were only three patients (1.2%) whose number of problems did not change across any of the ICF-DOC chapters. 

For the Body Functions domain (Table 2), the extension and severity of problems remained relatively stable for the majority of patients. However, chapters on which >20% of patients experienced an improvement were Sensory functions and pain (b2), Functions of the cardiovascular, haematological, immunological and respiratory systems (b4), Functions of the digestive, metabolic, endocrine systems (b5), Genitourinary and reproductive functions (b6) and Functions of the skin and related structures (b8) (extension index) and b6 and b8 (severity index). For the Body Structures domain (Table 3), the extension and severity indexes remained stable for the majority of patients. *A* > 20% improvement was found for the chapters Structures of the cardiovascular, immunological and respiratory systems (s4) and Structures related to movement (s7) (extension index) and s7 (severity index). Within the Activities & Participation domain (Table 4), in relation to performance, *a* > 20% improvement was found for the chapters Mobility (d4), Self care (d5), Domestic life (d6), and Community, social and civil life (d9) (extension index) and d4 and d6 (severity index). With respect to capacity, *a* > 20% improvement was found for the chapter d6 (severity index). Lastly, for the Environmental Factors domain (Table 5), the extension and severity of the facilitators remained relatively stable for the majority of patients. The extension and severity of the barriers remained highly stable. With regards to the facilitators, *a* > 20% improvement was observed for chapter Support and relationships (e3) (severity index). In relation to the barriers, there were no improvements >20% for any of the chapters. 

**Table 2:** Number and percentage of patients who improved, remained stable, or deteriorated with respect to the number of problems of any severity (extension index) or severe or complete problems (severity index) on the various chapters within the Body Functions domain.

ICF Chapter		Extension (1–4 & 8)						Severity (3–4)					
		Improvement		Stable		Deterioration		Improvement		Stable		Deterioration	
B Body Function Domain	Valid *N*	*N*	%	*N*	%	*N*	%	*N*	%	*N*	%	*N*	%
b1	174	28	16.1	116	66.7	30	17.2	34	19.5	98	56.3	42	24.1
b2	194	47	24.2	121	62.4	26	13.4	21	10.8	124	63.9	49	25.3
b3	200	11	5.5	188	94.0	1	0.5	13	6.5	181	90.5	6	3.0
b4	189	38	20.1	109	57.7	42	22.2	31	16.4	140	74.1	18	9.5
b5	208	48	23.1	117	56.3	43	20.7	35	16.8	112	53.8	61	29.3
b6	119	38	31.9	77	64.7	4	3.4	25	21.0	73	61.3	21	17.6
b7	217	29	13.4	159	73.3	29	13.4	43	19.8	133	61.3	41	18.9
b8	127	69	54.3	55	43.3	3	2.4	82	64.6	42	33.1	3	2.4
*B* total	175	68	38.9	30	17.1	77	44.0	67	38.3	44	25.1	64	36.6

Note: b1 Mental functions; b2 Sensory functions and pain; b3 Voice and speech functions; b4 Functions of the cardiovascular, haematological, immunological and respiratory systems; b5 Functions of the digestive, metabolic, endocrine systems; b6 Genitourinary and reproductive functions; b7 Neuromusculoskeletal and movement-related functions; b8 Functions of the skin and related structures; B Body Function Domain. Improvement = a decrease in the number of problems between T0 and T1, stability = the number of problems remained the same between T0 and T1, and deterioration = an increase in the number of problems between T0 and T1.

**Figure 2:**
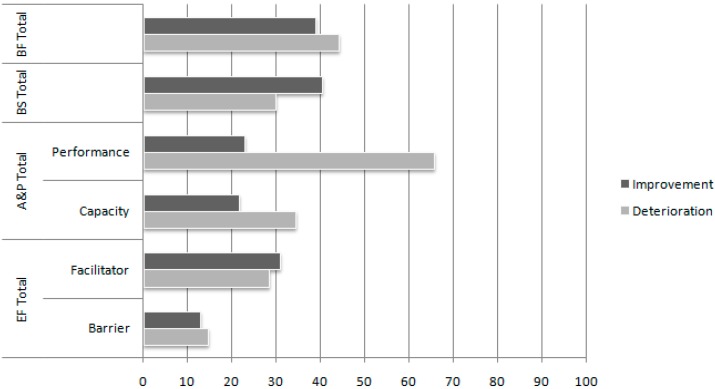
Comparison of the percentage of patients who improved or deteriorated on the various International Classification of Functioning, Disability, and Health domains with respect to the number of problems (extension index) between T0 and T1. The number of problems of the remaining patients did not change.

**Figure 3:**
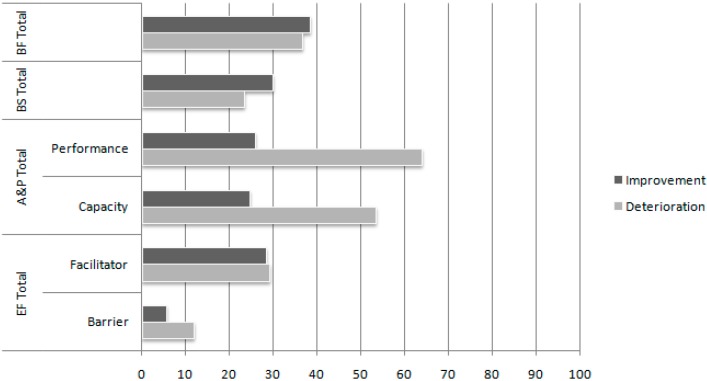
Comparison of the percentage of patients who improved or deteriorated on the various International Classification of Functioning, Disability, and Health domains with respect to the number of severe or complete problems (severity index) between T0 and T1. The number of problems of the remaining patients did not change.

Improvement and deterioration in extension and severity of problems in the total scores on the four ICF domains are presented in Figure 2 and Figure 3. Improvement was found for 38.9% (extension) and 38.3% (severity) of patients in the Body Functions domain, 40.3% (extension) and 29.9% (severity) in the Body Structures domain, 22.7% (extension) and 25.8% (severity) in performance and 21.5% (extension) and 24.5% (severity) in capacity in the Activities & Participation domain, and 28.2% (extension) and 29.1% (severity) in facilitators and 12.7% (extension) and 5.5% (severity) in barriers in the Environmental Factors domain. 

**Table 3:** Number and percentage of patients who improved, remained stable, or deteriorated with respect to the number of problems of any severity (extension index) or severe or complete problems (severity index) on the various chapters within the Body Structures domain.

ICF Chapter		Extension (1–4&8)						Severity (3–4)					
		Improvement		Stable		Deterioration		Improvement		Stable		Deterioration	
S Body Structure Domain	Valid *N*	*N*	%	*N*	%	*N*	%	*N*	%	*N*	%	*N*	%
s1	235	5	2.1	228	97.0	2	0.9	9	3.8	211	89.8	15	6.4
s3	144	12	8.3	121	84.0	11	7.6	4	2.8	133	92.4	7	4.9
s4	180	51	28.3	116	64.4	13	7.2	17	9.4	155	86.1	8	4.4
s5	247	6	2.4	241	97.6	0	0.0	0	0.0	244	98.8	3	1.2
s6	186	26	14.0	147	79.0	13	7.0	1	0.5	178	95.7	7	3.8
s7	149	44	29.5	69	46.3	36	24.2	36	24.2	87	58.4	26	17.4
s8	206	27	13.1	163	79.1	16	7.8	10	4.9	190	92.2	6	2.9
*S* total	154	62	40.3	46	29.9	46	29.9	46	29.9	72	46.8	36	23.4

Note: s1 Structure of the nervous system; s2 The eye, ear and related Structures; s3 Structures involved in voice and speech; s4 Structure of the cardiovascular, immunological and respiratory systems; s5 Structures related to the digestive, metabolic and endocrine systems; s6 Structure related to genitourinary and reproductive Systems; s7 Structure related to movement; s8 Skin and related structures; S Body Structure Domain. Improvement = a decrease in the number of problems between T0 and T1, stability = the number of problems remained the same between T0 and T1, and deterioration = an increase in the number of problems between T0 and T1.

**Table 4:** Number and percentage of patients who improved, remained stable, or deteriorated with respect to the number of problems of any severity (extension index) or severe or complete problems (severity index) on the various chapters within the Activities & Participation domain.

ICF Chapter		Extension (1–4&8)												Severity (3–4)											
		Performance						Capacity						Performance						Capacity					
		Improvement		Stable		Deterioration		Improvement		Stable		Deterioration		Improvement		Stable		Deterioration		Improvement		Stable		Deterioration	
D Activities & Participation Domain	Valid *N*	*N*	%	*N*	%	N	%	N	%	*N*	%	*N*	%	*N*	%	*N*	%	N	%	*N*	%	*N*	%	*N*	%
d1	157	9	5.7	145	92.4	3	1.9	9	5.7	143	91.1	5	3.2	24	15.3	122	77.7	11	7.0	15	9.6	124	79.0	18	11.5
d2	177	6	3.4	171	96.6	0	0.0	3	1.7	174	98.3	0	0.0	15	8.5	160	90.4	2	1.1	8	4.5	168	94.9	1	0.6
d3	183	8	4.4	158	86.3	17	9.3	6	3.3	160	87.4	18	9.8	23	12.6	140	76.5	20	10.9	16	8.7	146	79.8	22	12.0
d4	158	37	23.4	29	18.4	92	58.2	4	2.5	138	87.3	17	10.8	35	22.2	49	31.0	74	46.8	13	8.2	129	81.6	17	10.8
d5	244	78	32.0	85	34.8	81	33.2	6	2.5	225	92.2	11	4.5	8	3.3	183	75.0	53	21.7	11	4.5	221	90.6	10	4.1
d6	223	45	20.2	86	38.6	92	41.3	38	17.0	182	81.6	3	1.3	46	20.6	89	39.9	88	39.5	39	17.5	181	81.2	3	1.3
d7	168	13	7.7	142	84.5	13	7.7	8	4.8	151	89.9	10	6.0	25	14.9	126	75.0	17	10.1	13	7.7	145	86.3	11	6.5
d8	144	16	11.1	64	44.4	64	44.4	10	6.9	118	81.9	18	12.5	19	13.2	64	44.4	61	42.4	12	8.3	115	79.9	19	13.2
d9	158	33	20.9	123	77.8	2	1.3	22	13.9	134	84.8	1	0.6	15	9.5	78	49.4	65	41.1	9	5.7	73	46.2	75	47.5
*D* total	163	37	22.7	19	11.7	107	65.6	35	21.5	71	43.6	56	34.4	42	25.8	17	10.4	104	63.8	40	24.5	35	21.5	87	53.4

Note: d1 Learning and applying knowledge; d2 General tasks and demands; d3 Communication; d4 Mobility; d5 Self care; d6 Domestic life; d7 Interpersonal interactions and relationships; d8 Major life areas; d9 Community, social and civil life; D Activities & Participation Domain . N varies due to missing values on the individual ICF categories. Improvement = a decrease in the number of problems between T0 and T1, stability = the number of problems remained the same between T0 and T1, and deterioration = an increase in the number of problems between T0 and T1.

**Table 5:** Number and percentage of patients who improved, remained stable, or deteriorated with respect to the number of facilitators and barriers (extension and severity indexes) on the various chapters within the Environmental Factors domain.

ICF Chapter		Extension (1–4&8)												Severity (3–4)											
		Facilitator						Barrier						Facilitator						Barrier					
E Environmental Factors Domain	Valid *N*	Improvement		Stable		Deterioration		Improvement		Stable		Deterioration		Improvement		Stable		Deterioration		Improvement		Stable		Deterioration	
		*N*	%	*N*	%	*N*	%	*N*	%	*N*	%	*N*	%	*N*	%	*N*	%	*N*	%	*N*	%	*N*	%	*N*	%
e1	97	6.0	6.2	69.0	71.1	22	22.7	3	3.1	85	87.6	9	9.3	8	8.2	72	74.2	17	17.5	1	1.0	88	90.7	8	8.2
e2	132	8.0	6.1	117.0	88.6	7	5.3	2	1.5	130	98.5	0	0.0	9	6.8	120	90.9	3	2.3	1	0.8	131	99.2	0	0.0
e3	121	24.0	19.8	82.0	67.8	15	12.4	6	5.0	108	89.3	7	5.8	25	20.7	77	63.6	19	15.7	3	2.5	114	94.2	4	3.3
e4	97	18.0	18.6	58.0	59.8	21	21.6	13	13.4	76	78.4	8	8.2	16	16.5	58	59.8	23	23.7	3	3.1	89	91.8	5	5.2
e5	94	13.0	13.8	62.0	66.0	19	20.2	3	3.2	80	85.1	11	11.7	16	17.0	62	66.0	16	17.0	2	2.1	86	91.5	6	6.4
*E* total	110	31	28.2	45	40.9	34	30.9	14	12.7	80	72.7	16	14.5	32	29.1	47	42.7	31	28.2	6	5.5	91	82.7	13	11.8

Note: e1 Products and technology; e2 Natural environment and human-made changes to environment; e3 Support and relationships; e4 Attitudes; e5 Services, systems and policies; E Environmental Factors Domain. For facilitators: improvement = an increase in the number of facilitators between T0 and T1, stability = the number of facilitators remained the same between T0 and T1, and deterioration = a decrease in the number of facilitators between T0 and T1. For barriers: improvement = a decrease in the number of barriers between T0 and T1, stability = the number of problems remained the same between T0 and T1, and deterioration = an increase in the number of barriers between T0 and T1.

### 3.2. Relative Variability in Functioning and Disability in Acute and Chronic Patients

A comparison of the relative variability in the various aspects of functioning and disability as defined by ICF between T0 and T1 is presented in Table 6. Overall, relative variability was higher for acute patients than for chronic ones, indicating that the number of problems of acute patients was more susceptible to change than that of chronic ones. The number of problems in the Body Functions domain decreased in acute patients and also slightly decreased in chronic patients. Interestingly, within the Activities & Participation domain, the number of problems somewhat decreased in acute patients, whereas in chronic patients, an increase was found, although this difference did not reach statistical significance. The only significant difference in relative variability between the two groups was found for chapter e3 Support and relationships (*Z* = − 2.75, *p* = 0.006, Cohen’s *d* = 0.78), reflecting the relatively large increase in facilitators pertaining to this chapter in acute patients (relative variability = 34.13), whereas the number of facilitators in chronic patients remained stable (relative variability = 1.30). With regards to the other chapters within the Environmental Factors domain, a decrease in the number of facilitators was observed in acute patients, with the largest decrease found in the chapter Natural environment and human-made changes to environment (e2) (relative variability = −23.08), whereas chronic patients showed a decrease in the number of facilitators in the chapters Products and technology (e1) and e2 and small increases in the chapters Attitudes (e4) and Services, systems and policies (e5). 

**Table 6:** Comparison of relative variability in International Classification of Functioning, Disability, and Health domains in acute *vs.* chronic patients.

ICF Aspect	Acute Patients (<1 year)		Chronic Patients (>1 year)		Difference
	*N*	*RV* (mean)	*N*	*RV* (mean)	*p*
BF	28	−6.15	147	−0.05	0.097
D (perf)	26	−2.45	137	11.92	0.086
e1 (fac)	20	−10.42	77	−2.60	0.113
e2 (fac)	13	−23.08	52	−4.81	0.295
e3 (fac)	21	34.13	99	1.30	0.006 ^*^
e4 (fac)	17	−3.92	68	4.98	0.787
e5 (fac)	17	−6.48	76	0.16	0.093

Note: *RV* indicates mean relative variability in scores between T0 and T1. Differences between acute and chronic patients were examined using Mann Whitney *U* tests. After Bonferroni correction, significance level was set at 0.00714. ^*^ indicates significance.

### 3.3. Predictors of Relative Variability in Activities *&* Participation

Results of the regression analysis, in which relative variability in Activities & Participation performance was regressed on age at the time of an acute event, time from event (at T0), and relative variability in the Body Functions domain and the chapters of the Environmental Factors domain, are presented in Table 7. Age at event, time from event, and relative variability in the Environmental Factors domain were found to be significant predictors of relative variability in the Activities & Participation domain. The negative relationship between age at event and relative variability in Activities & Participation performance (β = −0.196, *t* = −2.080, *p* = 0.040) indicates that the older a patient is at the time of the acute event, the lower his relative variability is in the number of problems in Activities & Participation performance domain between T0 and T1. 

Similarly, the negative relationship between time from event (assessed at T0) and relative variability in Activities & Participation performance (β = −0.234, *t* = −2.532, *p* = 0.013) shows that the longer the time from the acute event is, the lower is the patient’s relative variability in the number of problems with respect to Activities & Participation performance between T0 and T1. 

Finally, the negative association between relative variability in the Environmental Factors domain and the relative variability in the Activities & Participation performance domain (β = −0.193, *t* = −2.007, *p* = 0.047) suggests that the more relative variability a patient has in the Environmental Factors domain, the lower his relative variability is in the number of problems within the Activities & Participation performance domain. Overall, the regression model explained 17.7% of the variance (*R* = 0.421, *R*^2^ = 0.177, *p* = 0.001). 

**Table 7:** Predictors of relative variability in Activities & Participation (performance).

Predictors	*B*	β	*t*	*p*
Age at event	−0.196	−0.196	−2.080	0.040 ^*^
Time from event (T0)	−0.234	−0.234	−2.532	0.013 ^*^
*RV* Body Functions domain	0.135	0.135	1.384	0.169
*RV* Environmental Factors domain	−0.193	−0.193	−2.007	0.047 ^*^

Note: *RV* = relative variability. *R* = 0.421, *R*^2^ = 0.177, *p* = 0.001.

## 4. Discussion

The aim of the current study was to examine longitudinal changes in disability and functioning in patients with disorders of consciousness using the ICF-DOC based on the biopsychosocial model. 

The main results of the study were the following. Only three patients in the whole sample did not fluctuate in their functionality on any of the ICF-DOC chapters between T0 and T1. Although the majority of patients remained stable or deteriorated in terms of the number of problems they experienced, there were also subgroups of patients who did show an improvement in functioning over time. Improvement with respect to the total scores on the four ICF domains was observed in approximately a quarter to half of the sample, depending upon the domain, except for barriers within the Environmental Factors domain, in which only a small minority of patients showed an improvement. These results show that there was generally some variability in functioning over time, leading us to conclude that our sample of patients with disorders of consciousness is not as stable as is often believed to be for these patients. Even when diagnosis remains the same, functioning can change over time. In addition, differences in changes between extension and severity indexes were relatively small, indicating that susceptibility to change over time was not dependent upon the severity of the problem. These findings challenge the position that is often taken by the insurance industry, which classifies patients with prolonged disorders of consciousness as “*untreatable*” [34], in the sense that rehabilitation and treatment for disorders of consciousness are not recognized because of the uncertainty of their prognosis and the fact that the prospects for clinically meaningful recovery are considered unlikely [9,27]. As a result, proactive, aggressive interventions for this population are mostly unavailable [34]. Hopefully, this situation will change for the better in the near future. Although definitive claims regarding the benefits of rehabilitation are still lacking [27], there is accumulating evidence that intensive rehabilitative interventions may have a positive effect on functional outcomes in patients with disorders of consciousness [25,26,35,36,37,38,39,40]. The ICF-DOC may be a suitable instrument to assess such changes in functional outcomes, as it may capture changes in aspects of functioning that are overlooked by classic clinical disability and function scales which do not take environmental factors into account [24,25]. 

Secondly, results of a comparison of relative variability in the various aspects of the ICF between T0 and T1 revealed that, overall, relative variability was higher for acute patients than for chronic ones, indicating that the number of problems of acute patients was more susceptible to change than the number of problems experienced by chronic patients. Interestingly, the number of problems within the Activities & Participation domain decreased somewhat in acute patients, whereas an increase was found in chronic patients, indicating that, especially in the longer term, patients with disorders of consciousness are at risk of deteriorations in Activities & Participation. These findings are in line with previous research reporting that, for the majority of patients, improvement, if any, usually occurs within the first six months to one year following the event [3,41], although recent studies have shown that recovery beyond these classic time limits is possible in up to 20% of patients [25,26,27,29,31,42,43,44], indicating that late recovery is not as exceptional as previously thought [45]. 

The only significant difference in relative variability between the two groups, which had a medium to large effect, was found for the environmental factor “*Support and relationships*”, reflecting the relatively large increase in facilitators pertaining to this chapter in acute patients, whereas the number of facilitators in chronic patients remained stable. These results are in line with previous findings regarding the crucial role of family members in the care for patients with disorders of consciousness [46]. However, special attention should be paid to caregivers’ health: recent studies [47,48,49] highlighted that caregivers of patients with disorders of consciousness show high levels of burden over time. Considering the fact that in many countries, including Italy, health policies promote at home care of patients with disorders of consciousness for long term care, the involvement of caregivers in the care process should be supported by tailored health programmes in order to prevent problems for patients’ and also for caregivers’ wellbeing. 

Thirdly, results showed that age at event, time from event, and relative variability in the Environmental Factors domain were significant predictors of relative variability in the Activities & Participation performance domain. The negative relationships between age at event and time from event and relative variability in Activities & Participation indicates that older patients and those who have unresponsive wakefulness syndrome or are in minimally conscious state for a longer period of time are less susceptible to experience changes in the Activities & Participation performance domain. These results are in line with previous studies reporting that younger patients with traumatic aetiology have a better prognosis [28,50], whereas older patients with non-traumatic aetiology show a lower functional recovery rate [43] and that a longer time from the event is usually associated with poorer outcomes [34]. Lastly, the negative association between relative variability in the Environmental Factors domain and relative variability in the Activities & Participation domain specifies that the more relative variability a patient has in the Environmental Factors domain, the lower his relative variability within the Activities & Participation domain. In terms of interpretation, this finding shows that if factors within the patient’s environment that previously facilitated his/her functioning decrease or disappear over time, patients with disorders of consciousness experience an increase in the number of problems in Activities & Participation performance domains. From a public health perspective, this result implies that health systems have to be cognizant of the long-term needs of patients with disorders of consciousness, and need to take positive steps in order to prevent a further deterioration in their functioning. Overall, the regression model explained a reasonable amount of the variance in Activities & Participation, leaving room for future studies to explore additional factors that may play a role in the prediction of changes in Activities & Participation over time. 

It is difficult to compare these results to other longitudinal studies in terms of functional outcomes in patients with disorders of consciousness, due to the earlier described differences between studies with respect to diagnostic criteria, setting, global region of health care provision, study sample, follow-up duration, and outcomes evaluated [26,30]. As a consequence, results are understandably disparate across studies. Some studies reported rather positive outcomes. For example, a study by Eilander and colleagues [25] found that, although full recovery was rare, the majority of patients eventually reached a semi-independent level of functioning. Among those with traumatic aetiology, about one third was moderately independent at follow-up, one third was mildly or moderately dependent, and one third was noticeably or totally dependent, whereas among those with non-traumatic aetiology the majority (64%) was noticeably or totally dependent. Another recent study by Katz and colleagues [27] reported that 27% were partially or mildly disabled at follow-up, 41% were moderately to severely disabled, and 32% were severely or extremely severely disabled. Nearly half of the patients achieved recovery to safe, daytime independence at home and 22% returned to work or school within two years after injury. By contrast, a study by Luauté and colleagues [28] reported very poor functional outcomes. Approximately 60% of patients in minimally conscious state either remained in this condition or died during the five-year follow-up period. Among those who regained consciousness, all remained severely disabled with significant motor and cognitive sequelae. Likewise, Estraneo and colleagues [29] found that both patients with and without traumatic aetiology who recovered consciousness remained severely to extremely severely disabled. A recent study by Eilander and colleagues [26] reported that, at follow-up, most patients were moderately disabled. Of the seven patients with unresponsive wakefulness syndrome at discharge, four had died at follow-up and the remaining three patients were very severely disabled. Finally, a recent study by Seel *et al.*, [31] reported that patients with disorders of consciousness continued to require a high level of medical care after specialized early treatment, although they showed significant improvements in terms of conscious functioning, respiratory function, hypertonia, pressure ulcers, and self-care activities. To conclude, as, with the exception of the latter study [31], none of these other studies examined functional outcome using the ICF, it is difficult to directly compare results, however, most studies reported significant levels of disability in patients with disorders of consciousness, like those found in the current study. More conformity in study design and methodology in future studies would contribute to better comparability between study results. 

Some limitations of the current study should be noted. First, the substantial amount of missing values at baseline significantly reduced the effective sample size to be used in the analyses. The high number of missing values should probably be attributed to the large number of instruments administered during the baseline assessment. However, corrective measures before and during data collection, such as more explicit instructions to those conducting the data collection as well as regular quality checks, decreased the number of missing values at follow-up, proving that these techniques were successful. Next, although this was not one of the primary aims of the current study, the level of specificity of the data regarding rehabilitation did not allow us to make inferences about the association between specific types of rehabilitation followed by the patients and longitudinal changes in ICF. This was due to the lack of information regarding the duration and intensity of the therapies, the fact that the majority of patients followed different combinations of multiple therapies which were provided by different centers, impairing comparability between them [25], and also a considerable variation in time from event between patients. In addition, for some patients, information regarding rehabilitation was not available. A final limitation that should be explicitly reported is that in the current study, disorders of consciousness have been taken as a broad category that includes both patients with unresponsive wakefulness syndrome and those in a minimally conscious state, as well as acute and chronic states of both conditions. It was decided to analyze data of the total sample because we wanted to describe functioning and disability associated to disorders of consciousness (defined as a syndrome with varying degrees of impairment). As described by Sattin *et al.*, in 2014 [7], level of functioning, not diagnosis, is the core concept for rehabilitation of patients with disorders of consciousness considering the biopsychosocial model represented by the ICF. As a consequence, our results do not distinguish between subcategories of patients with disorders of consciousness, which may differ in terms of impairment of functioning. It is thus possible that the described longitudinal changes mainly reflect changes in patients in a minimally conscious state, and not those with unresponsive wakefulness syndrome. Although studies have pointed at the high rate of diagnostic inaccuracy in diagnosing unresponsive wakefulness syndrome *vs.* minimally conscious state [16,51,52,53], making it more difficult to accurately compare outcomes between these groups, and others have stressed the importance of other factors, such as aetiology, over diagnostic category [54], we acknowledge that patients with unresponsive wakefulness syndrome in general have been found to have a worse prognosis [26,28,55], especially those who have been classified as “*persistent*” or “*permanent*” [29]. We therefore encourage future studies to conduct further, more specific analyses of longitudinal changes in ICF in subcategories of patients with disorders of consciousness. 

This study also has several strengths. First, data collection has been conducted in 90 different Italian centers all over the country, and thus the sample can be considered an adequate representation of the general population of patients with disorders of consciousness in Italy. Another strength of this study is its sample size of 248, which can be considered large given the clinical nature of the population. A final strength of this study concerns its longitudinal design. To our knowledge, this is one of the first longitudinal ICF-based study in patients with disorders of consciousness. Other studies have stressed the need for multicenter longitudinal studies on large cohorts of patients [29] that provide a more fine-grained description of patients with disorders of consciousness in terms of their capabilities and impairments [28]. 

Future studies are recommended to replicate and extend these results in other samples, preferably using multiple measurement occasions. Ideally, the ICF-DOC would be administered regularly (e.g., once a year) to systematically monitor biopsychosocial functioning and disability in patients with disorders of consciousness. Routine reexamination of functioning in these patients is fundamental to evaluate the effects of interventions [24] and to inform the crucial decisions made for these patients [9,42]. Moreover, systematic monitoring of functioning in patients with disorders of consciousness using the ICF-DOC would enable the development of normative values in order to assess whether changes in functioning are also clinically meaningful, in addition to statistical significance. Ongoing scientific and clinical developments regarding the care for patients with disorders of consciousness raise several important public health-related issues. A first important public health priority relates to the urgent need for standardized guidelines for diagnosis and treatment and, consequently, better access to care [17]. The lack of standardized clinical guidelines for patients in a minimally conscious state and the outdated guidelines for patients with unresponsive wakefulness syndrome that do not consider proactive treatment approaches have important consequences for the treatment and management of patients with disorders of consciousness. For example, US insurance companies do not recognize treatment for disorders of consciousness, and thus interventions aimed at recovery of functioning are often unavailable or inaccessible [34]. A recent Dutch study [56] also found that patients with disorders of consciousness have poor access to rehabilitation. As a result, these patients are often placed in long-term care facilities or sent home, where they receive merely palliative care [34]. This situation puts an enormous burden on the patient’s family, the community, and the public health systems [34]. Often experiencing feelings of sadness and grief, a loss of income as a consequence of caring duties, high medical costs, and difficult ethical dilemmas regarding the patient’s treatment, and issues such as withdrawal of artificial nutrition and hydration, it does not come as a surprise that caregivers of patients with disorders of consciousness show elevated levels of burden and distress over time [47,48,49]. It remains an important public health responsibility to provide tailored support to caregivers over time in order to ensure adequate care of patients with disorders of consciousness. A recent study [31] suggested that families who receive comprehensive education and training with continuing follow-up support may be better prepared to provide at home care for their relatives. Also, health professionals involved in the care for patients with disorders of consciousness appear to experience elevated levels of discomfort and distress [9]. Caring for these patients can lead to ambiguous emotions and conflicting views on their appropriate medical care [57,58]. Strategies should be developed to prevent potential miscommunications and sources of misunderstandings between all parties involved in the diagnosis, treatment and care of patients with disorders of consciousness [9]. A final important public health challenge lies in the education of the general public as well as the further training of healthcare professionals working with patients with disorders of consciousness [9,17]. Studies have shown that unresponsive wakefulness syndrome and minimally conscious state are conditions that are not well understood by the general population [59], and that exceptional or high-profile cases in the media bias the general impression of disorders of consciousness [60,61,62]. Health professionals working with patients with disorders of consciousness could benefit from institutional educational programs on clinical aspects of disorders of consciousness and the associated ethical challenges [9].

## 5. Conclusion

Patients with disorders of consciousness are by definition completely dependent upon their environment. In this study, we conclude that it is important that the environment of these patients is maintained and adapted to support and facilitate them, especially in the long term, in order to prevent an inevitable decrease in their functioning and, subsequently, an increase in their disability. It remains an important public health responsibility of policy makers to ensure tailored support to caregivers over time in order to continue to provide appropriate care to patients with disorders of consciousness who show low levels of functioning and high needs of environmental facilitators. 

## Supplementary Files

Supplementary File 1
