# Supplementary material for: Longitudinal Changes in Functioning and Disability in Patients with Disorders of Consciousness: The Importance of Environmental Factors
Source: Int J Environ Res Public Health. 2015 Apr 1;12(4):3707–30. doi: 10.3390/ijerph120403707 (PMC4410211; doi:10.3390/ijerph120403707)
Supplement: Supplementary File 1 [file ijerph-12-03707-s001.pdf]

Supplementary Information

Longitudinal Changes in Functioning and Disability in Patients with Disorders of Consciousness: The Importance of Environmental Factors

---

Table S1. Overview of ICF-DOC dedicated checklist for patients with disorders of consciousness.

Code	Body Functions	Code	Body Structures	Code	Activities & Participation	Code	Environmental Factors
b110	Consciousness functions	s110	Structure of brain	d110	Watching	e110	Products or substances for personal consumption
b114	Orientation functions	s3	Structures involved in voice and speech	d115	Listening	e115	Products and technology for personal use in daily living
b117	Intellectual functions			d175	Solving problems	e120	Products and technology for personal indoor and outdoor mobility and transportation
b130	Energy and drive functions	s410	Structure of cardiovascular system	d210	Undertaking a single task		
b134	Sleep functions			d220	Undertaking a multiple tasks	e125	Products and technology for communication
b140	Attention functions	s430	Structure of respiratory system	d310	Communicating with-receiving-spoken messages	e150	Design, construction and building products and technology of buildings for public use
b144	Memory functions	s530	Structure of stomach				
b152	Emotional functions	s610	Structure of urinary system	d315	Communicating with-receiving-nonverbal messages	e155	Design, construction and building products and technology of buildings for private use
b156	Perceptual functions	s710	Structure of head and neck region				
b164	Higher-level cognitive functions			d330	Speaking	e225	Climate
b167	Mental functions of language	s720	Structure of shoulder region	d335	Producing nonverbal messages	e240	Light
b210	Seeing functions	s730	Structure of upper extremity	d350	Conversation	e250	Sound

Table S1. *Cont.*

Code	Body Functions	Code	Body Structures	Code	Activities & Participation	Code	Environmental Factors
b230	Hearing functions	s740	Structure of pelvic region	d415	Maintaining a body position	e310	Immediate family
b235	Vestibular functions	s750	Structure of lower extremity	d420	Transferring oneself	e320	Friends
b280	Sensation of pain	s760	Structure of trunk	d430	Lifting and carrying objects	e325	Acquaintances, peers, colleagues, neighbors and community members
b310	Voice functions	s810	Structure of areas of skin	d440	Fine hand use		
b410	Heart functions			d450	Walking	e330	People in positions of authority
b420	Blood pressure functions			d460	Moving around in different locations	e340	Personal care providers and personal assistants
b435	Immunological system functions			d465	Moving around using equipment	e355	Health professionals
b440	Respiration functions			d470	Using transportation	e360	Other professionals
b445	Respiratory muscle functions			d475	Driving	e410	Individual attitudes of immediate family members
b450	Additional respiratory functions			d510	Washing oneself	e420	Individual attitudes of friends
b510	Ingestion functions			d520	Caring for body parts	e440	Individual attitudes of personal care providers and personal assistants
b5105	Swallowing			d530	Toileting	e450	Individual attitudes of health professionals
b515	Digestive functions			d540	Dressing	e455	Individual attitudes of other professionals
b525	Defecation functions			d550	Eating	e460	Societal attitudes
b5253	Faecal continence			d560	Drinking	e465	Social norms, practices and ideologies
b530	Weight maintenance functions			d570	Looking after one's health	e525	Housing services, systems and policies
b620	Urination functions			d620	Acquisition of goods and services	e535	Communication services, systems and policies
b6202	Urinary continence			d630	Preparing meals	e540	Transportation services, systems and policies
b640	Sexual functions			d640	Doing house work	e550	Legal services, systems and policies

Table S1. *Cont.*

Code	Body Functions	Code	Body Structures	Code	Activities & Participation	Code	Environmental Factors
b710	Mobility of joint functions			d660	Assisting others	e555	Associations and organizational services, systems and policies
b730	Muscle power functions			d710	Basic interpersonal interactions	e570	Social security services, systems and policies
b735	Muscle tone functions			d7106	Differentiation of family persons	e575	General social support services, systems and policies
b760	Control of voluntary movement functions			d720	Complex interpersonal interactions	e580	Health services, systems and policies
b765	Involuntary movement functions			d730	Relating with strangers		
b8	Functions of the skin			d740	Formal relationships		
				d750	Informal social relationships		
				d760	Family relationships		
				d770	Intimate relationships		
				d810	Informal education		
				d850	Remunerative employment		
				d860	Basic economic transactions		
				d870	Economic self-sufficiency		
				d910	Community life		
				d920	Recreation and leisure		
				d930	Religion and spirituality		
				d950	Political life and citizenship		

© 2015 by the authors; licensee MDPI, Basel, Switzerland. This article is an open access article distributed under the terms and conditions of the Creative Commons Attribution license (http://creativecommons.org/licenses/by/4.0/).
